# NGS for (Hemato-) Oncology in Belgium: Evaluation of Laboratory Performance and Feasibility of a National External Quality Assessment Program

**DOI:** 10.3390/cancers12113180

**Published:** 2020-10-29

**Authors:** Thomas Delcourt, Kevin Vanneste, Mohamed Rida Soumali, Wim Coucke, Vanessa Ghislain, Aline Hebrant, Els Van Valckenborgh, Sigrid C. J. De Keersmaecker, Nancy H. Roosens, Philippe Van De Walle, Marc Van Den Bulcke, Aline Antoniou

**Affiliations:** 1 Transversal activities in Applied Genomics, Sciensano, 1050 Brussels, Belgium; Thomas.Delcourt@sciensano.be (T.D.); Kevin.Vanneste@sciensano.be (K.V.); Sigrid.DeKeersmaecker@sciensano.be (S.C.J.D.K.); Nancy.Roosens@sciensano.be (N.H.R.); 2 Quality of Laboratories, Sciensano, 1050 Brussels, Belgium; MohamedRida.Soumali@sciensano.be (M.R.S.); Wim.Coucke@sciensano.be (W.C.); Vanessa.Ghislain@sciensano.be (V.G.); Philippe.VanDeWalle@sciensano.be (P.V.D.W.); 3 Cancer Centre, Sciensano, 1050 Brussels, Belgium; Aline.Hebrant@sciensano.be (A.H.); Els.VanValckenborgh@sciensano.be (E.V.V.); Marc.VanDenBulcke@sciensano.be (M.V.D.B.)

**Keywords:** next-generation sequencing, hemato-oncology, oncology, external quality assessment, cancer

## Abstract

**Simple Summary:**

In recent years, high-throughput sequencing has been routinely used by medical laboratories to search for somatic mutations in (hemato-)oncology as diagnostic, prognostic or therapeutic markers in various cancers. Since 2016, Belgium has developed a comprehensive program to facilitate the implementation of this technology in the national healthcare system, requiring, among others, an external quality assessment (EQA) of laboratories using this technology. Three benchmarking trials were organized between 2017 and 2018, covering different pathologies to establish the state of the art of the current practices of the Belgian laboratories and prepare future EQA. This study has highlighted areas of improvement for laboratories and will serve as a baseline for the establishment of a sustainable national EQA.

**Abstract:**

Next-generation sequencing (NGS) is being integrated into routine clinical practice in the field of (hemato-) oncology to search for variants with diagnostic, prognostic, or therapeutic value at potentially low allelic frequencies. The complex sequencing workflows used require careful validation and continuous quality control. Participation in external quality assessments (EQA) helps laboratories evaluate their performance and guarantee the validity of tests results with the ultimate goal of ensuring high-quality patient care. Here, we describe three benchmarking trials performed during the period 2017–2018 aiming firstly at establishing the state-of-the-art and secondly setting up a NGS-specific EQA program at the national level in the field of clinical (hemato-) oncology in Belgium. DNA samples derived from cell line mixes and artificially mutated cell lines, designed to carry variants of clinical relevance occurring in solid tumors, hematological malignancies, and *BRCA1/BRCA2* genes, were sent to Belgian human genetics, anatomic pathology, and clinical biology laboratories, to be processed following routine practices, together with surveys covering technical aspects of the NGS workflows. Despite the wide variety of platforms and workflows currently applied in routine clinical practice, performance was satisfactory, since participating laboratories identified the targeted variants with success rates ranging between 93.06% and 97.63% depending on the benchmark, and few false negative or repeatability issues were identified. However, variant reporting and interpretation varied, underlining the need for further standardization. Our approach showcases the feasibility of developing and implementing EQA for routine clinical practice in the field of (hemato-) oncology, while highlighting the challenges faced.

## 1. Introduction

Next-generation sequencing (NGS) has revolutionized DNA sequencing by allowing the retrieval of massive amounts of information from biological material, especially compared to traditional methods such as Sanger sequencing [1]. By means of targeted sequencing, this power can be leveraged to obtain high-depth sequencings of specific genomic regions at a relatively low cost per base [2]. This is, amongst others, particularly effective in the field of (hemato-) oncology where there exists a need to identify specific variants of clinical relevance (whether germline or somatic) with prognostic, diagnostic, or therapeutic value at potentially very low frequencies within a list of known targets [3]. Therefore, laboratories performing routine cancer analysis have actively been adopting NGS-based assays over the last few years, both within Belgium [4] and elsewhere [5,6,7,8,9,10], paving the way toward personalized precision medicine [11].

Ensuring the competency of laboratories performing patient samples analysis [12] and ultimately guaranteeing standardized high-quality patients tests results is of paramount importance. However, different NGS technologies and platforms exist, each with their own inherent characteristics, limitations, and advantages [13], but all are typically characterized by a lengthy and complex workflow to go from sample to result, requiring multiple steps including sample preparation, library preparation, sequencing, and bioinformatics analysis, which each have the potential to introduce sources of error and variation [14].

Within Belgium, the introduction of NGS in the healthcare system for (hemato-) oncology is formulated in the “Roadbook for the implementation of next-generation sequencing in clinical practice in oncology and hemato-oncology in Belgium” [15], which concretizes it into 10 specific actions. In an effort to standardize the use of NGS, specific guidelines have been published by the Commission for Personalized Medicine (ComPerMed) (action 2) [16]. To evaluate specifically the added value of NGS in (hemato-) oncology and its integration within the national reimbursement system for healthcare, a convention between laboratories performing (hemato-) oncology testing and the National Institute for Health and Disability (INAMI/RIZIV, https://www.riziv.fgov.be), was set up as a pilot phase for the period 2019–2022 (action 9) [15]. This convention defines all the conditions to be fulfilled by the laboratories to obtain a reimbursement of NGS tests, among others the regions to target for each type of tested cancer [17]. Additionally, the performance of laboratories using NGS for routine cancer diagnostics in Belgium as well as the state-of-the-art of the field (actions 4 and 5) had to be established in order to ensure that they are capable of delivering high-quality test results.

As is commonplace in other medical domains [18] and required by the Belgian accreditation agency (BELAC, http://www.belac.fgov.be) [19], performing regular internal quality control (IQC) and participating in external quality assessment (EQA), also known as proficiency tests (PT), is considered an integral part of quality systems of medical laboratories that perform NGS-based tests, as this ensures the overall quality of provided services [12,20,21,22]. As part of the ISO15189 standard for medical laboratories accreditation [23], failures in EQAs and IQCs must be recorded as non-conformities in the laboratories quality system, which must then establish the origin of the error and formulate a preventive and corrective action plan, which is evaluated by BELAC during audits. While IQC requires the analysis of a priori known positive and negative samples, EQA requires well-characterized samples unknown to the laboratory to be analyzed according to their routine procedures.

Several international EQA providers have developed PT for somatic variants analysis, either disease- or gene-specific (in both of which cases, participants can use their preferred analytical method), or specific to NGS. Both Genomics Quality Assessment (GenQA, https://www.genqa.org) and the European Molecular Genetics Quality Network (EMQN, https://www.emqn.org) provide EQA for specific cancer types and have partnered to provide an annual NGS-specific EQA consisting of one sample of formalin-fixed paraffin-embedded (FFPE) genomic DNA material with a matching mock clinical case. The College of American Pathologists (CAP, https://www.cap.org) offers two bi-yearly shipments of three samples of DNA aimed at evaluating variants in respectively 28 and 24 genes involved in solid tumors or hematological malignancies. The European Society for Pathology (ESP, https://www.esp-pathology.org) provides disease-specific EQA for colorectal cancers and non-small cell lung carcinoma (NSCLC) based on FFPE tumor slides. Several national initiatives also currently exist or have taken place previously, such as the Dutch Foundation for Quality Assessment in Medical Laboratories (SKML, www.skml.nl), which has provided EQA for melanomas, colon, and lung cancers [12] and provides EQA for hematological malignancies, three EQA rounds produced in France between 2012 and 2014 that targeted metastatic colorectal cancer (CRC) and NSCLC [24], and an Italian EQA targeting CRC in 2015 [20].

No unique EQA currently provided internationally meets the requirements of the Belgian healthcare system in terms of genes covered, cancer types covered, and number of yearly shipments, nor are they guaranteed to do so in the future as the field evolves, and combining NGS-specific EQAs to allow covering a wide range of genes would make the cost of participation for Belgian laboratories prohibitive. Therefore, to allow regular monitoring and evaluation of the quality of NGS analyses performed in routine cancer diagnostics within Belgium, EQA will have to be implemented at the national level. To allow both evaluation of the current state-of-the-art and developing future EQA, three benchmarks were organized and evaluated by Sciensano (https://www.sciensano.be) during the period 2017–2018, which were open to all laboratories providing NGS-based cancer testing in Belgium and were accompanied by a state-of-the-art survey. These benchmarks were performed in collaboration with experts from the fields in which tumor sequencing is routinely carried out (pathology, genetics, and clinical biology) to ensure the incorporation of feedback from leading experts and to allow optimization of the quality assessment framework with the aim of implementing a national Belgian EQA system starting in 2021. To reflect the clinical reality of evaluated laboratories in Belgium, these benchmarks were separated in three activity domains usually covered by different laboratories and were designed to target major cancer types analyzed in Belgium [25]. Benchmark 2017/1 was dedicated to solid tumors, benchmark 2017/2 was dedicated to hematological malignancies, and benchmark 2018/1 was specifically dedicated to *BRCA1/2* genes. Participants were sent DNA samples and were requested to analyze them in the same workflow as routine samples, albeit in triplicates, i.e., as three independent samples in the same run of sequencing to allow for an analysis of repeatability. Assessment of results focusing on reported protein-level variants was the cornerstone of individual laboratory performance evaluation. Variants were only considered for performance evaluation if they were reported by at least two-thirds of participants, were present in regions of interest as defined in the latest drafts of the Belgian convention for the reimbursement of NGS tests in the routine diagnostic of (hemato-) oncology [17] and were evidenced by digital-droplet PCR (dd-PCR) or whole exome sequencing (WES). These variants were considered as evaluative variants, i.e., variants that were used to provide participants with a common basis for comparison and would constitute the basis for individual performance evaluation in an EQA scheme. Variants present in regions of interest and validated by dd-PCR or WES but reported by less than two-thirds of participants were considered as informative variants, and were provided in the reports for information only. To obtain an overview of the state-of-the-art of NGS workflows employed in clinical (hemato-) oncology within Belgium, participants of every benchmark were requested to complete a technical survey inquiring about several aspects of their NGS workflows (sample types, NGS platforms, sequencing characteristics, gene panels, bio-informatics pipelines). For each benchmark, the methodology was reviewed by discussing areas of improvement with the experts group, thus proceeding with a learn-by-doing approach. Here, we present results from these three benchmarks, highlighting and detailing our approach during this process. The results of the Belgian state-of-the-art survey and performance of participating laboratories that provide NGS-based oncology testing in Belgium are presented and discussed below, as well as the feasibility and considerations for implementing a national EQA framework for clinical tests based on NGS for (hemato-) oncology.

## 2. Results

### 2.1. Overview of Answers to the State-Of-The-Art Surveys

#### 2.1.1. Laboratories and Sample Types

Laboratories for anatomic pathology constituted the majority of participants for the solid tumors benchmark 2017/1 (62.5% anatomic pathology, 25% clinical biology, and 12.5% human genetics), whereas participants in the hematological benchmark 2017/2 were mostly clinical biology laboratories (6.7% anatomic pathology, 73.3% clinical biology, and 20% human genetics), and participants in the *BRCA1/2* benchmark 2018/1 were mainly human genetics laboratories (16.7% anatomic pathology, 33.3% clinical biology, and 50% human genetics). An overview of sample types analyzed routinely by participants per benchmark is presented in Supplementary Table S1. FFPE tumor samples were the most prevalent for benchmarks 2017/1 and 2018/1, and fresh bone marrow and blood samples were the most prevalent for benchmark 2017/2. Other routinely processed sample types included frozen tissue, biopsies, fresh tissue, cytological and biological liquid, swabs, and circulating tumor DNA (ctDNA).

#### 2.1.2. NGS Platforms

For all three benchmarks, most participants reported having access to an Illumina sequencing platform (Miseq: 87.50%, 86.67%, and 83.33% for benchmarks 2017/1, 2017/2, and 2018/1, respectively; Hiseq: 6.25%, 6.67%, and 0%; Nextseq: 18.75%, 0%, and 8.33%), with a minority reporting using the Ion Torrent PGM (12.50%, 6.67%, and 0%, respectively). The Qiagen GeneReader was only reported as being used by 8.33% of participants of benchmark 2018/1. Some participants reported having access to more than one platform for benchmark 2017/1. Correspondingly, the majority of participants generated paired-end reads. Required reads lengths varied between 75 and 350 bp, with the interquartile range between 150 and 230 bp.

#### 2.1.3. Bioinformatics Softwares, Reported Variant Types, and Limits of Detection

A notable variety of software was reported as being used for bioinformatics analysis in all three benchmarks (see Supplementary Table S2). Software packages most often employed included SeqNext (JSI), VariantStudio (Illumina)—albeit not in benchmark 2018/1, and Sophia DDM (Sophia Genetics). Only a minority of participants reported employing in-house scripts/pipelines (typically based on BWA [26] for read alignment and GATK [27] for variant calling) over all three benchmarks. For all three benchmarks, all participants declared routinely reporting single-nucleotide polymorphisms (SNPs) and indels, and in one case also copy-number variations (CNVs) and translocations in *BRCA1* and *BRCA2* for benchmark 2018/1. The limit of detection (LOD) reported by participants ranged from 100 to 1000 reads, and from 1 to 10% allelic frequency, depending on the detectable variant type (see Supplementary Table S3). Some participants reported lowering the minimum required read depth for hotspots variants.

#### 2.1.4. Gene Panels and Enrichment Strategies

All three benchmarks displayed a wide variety of gene panels used by the different participants (see Supplementary Table S4). Half of the target panels used by participants of benchmark 2017/1 were custom-designed (8/16 reported panels), whereas most participants in benchmarks 2017/2 and 2018/1 reported using commercially available panels (respectively 5/15 and 1/12 custom-designed panel usage reported). A majority of participants reported using amplicon-based enrichment strategies, although a minority also reported employing probe-based enrichment strategies. An overview of the minimum quantity of DNA employed is presented in Supplementary Table S5, and it ranged from <10 ng to 1000 ng, with some participants reporting different quantities depending on the panel employed.

### 2.2. Overview of Benchmark Results

#### 2.2.1. General

A general overview of benchmarks characteristics and of samples is provided in Table 1 and Table 2, respectively. The Material and Methods section further details the benchmark’s design. A detailed overview of results for all evaluative and informative variants per benchmark is provided in Table 3 and Table 4, respectively, and a summarized overview of participant success rates for every benchmark is provided in Table 5. Overall, the results were consistently good for all three benchmarks with 97.63%, 96.61%, and 93.06% of evaluative variants reported for benchmarks 2017/1, 2017/2, and 2018/1, respectively, indicating a high level of agreement between participating laboratories. The somewhat lower score for benchmark 2018/1 can be attributed to a lower number of participants combined with one participant missing five out of six variants, thereby bringing the overall score down.

#### 2.2.2. Assessment of the Total Number of Reported Variants and Strategies for Defining Evaluative and Informative Variants

The number of reported variants varied between benchmarks. For all three benchmarks, on top of the variants specifically ordered and validated through ddPCR by the vendor, the sample material contained additional variants in regions of interest defined by the convention of the INAMI/RIZIV [17] that pre-existed in the cell lines, and were either endogenous or inserted. Since participants were not asked to report variants at predefined positions but specifically at any clinically relevant position for the type of tumor material under investigation (see Table 2), these additional variants were also typically reported by participants. The different strategies employed in handling them for the three benchmarks are described below.

For benchmark 2017/1, 12 variants were ordered (Supplementary Table S6) and also reported by at least two-thirds of participants, but four additional variants existed in the sample material at clinically relevant positions that were also reported by at least two-thirds of participants and which had been previously confirmed by WES on the cell lines used for DNA production and were therefore also taken up in the set of evaluative variants resulting in a total set of 16 (Table 3).

For benchmark 2017/2, 16 variants were ordered (Supplementary Table S6) and reported by at least two-thirds of participants (Table 3). Since the benchmark covered a much wider range of reportable regions, a much larger set of additional variants at clinically relevant positions were present in the sample material and correspondingly reported by varying numbers of participants. These additional variants could not be evidenced by WES data, as some of the cell lines used during material production lacked prior WES information. As a result of economic considerations, these additional variants could not be post hoc validated by ddPCR and were considered as informative only and were not further analyzed (Table 4).

For benchmark 2018/1, six variants were ordered (Supplementary Table S6); however, of these, only four were reported by at least two-thirds of participants and used as evaluative variants (Table 3), whereas the remaining two were not reported by a single participant and therefore omitted for evaluation. An additional 14 variants evidenced by WES data were reported by no participant and were not further analyzed. Additionally, because the benchmark specifically covered the *BRCA1* and *BRCA2* genes, a more limited set of four additional variants at clinically relevant positions were reported of which only two were reported by at least two-thirds of participants. Since for benchmark 2018/1, the sample material consisted of pure cell lines that had been validated through WES, these two variants were also considered as evaluative (Table 3), while the two variants reported by less than two-thirds of participants were considered as informative only (Table 4), resulting in a total set of six evaluative and two informative variants. Principal results for evaluative variants are presented in the next sections, whereas informative variants are presented in Table 4 for completeness only.

#### 2.2.3. Assessment of Evaluative Variants

For every benchmark, the set of evaluative variants was considered as the ground truth to which the results of benchmark participants should be compared. Any evaluative variant reported in none of the triplicates was considered as a false negative observation (see Section 2.2.5 for results on repeatability). Since the ground truth was composed entirely of clinically relevant variants that had specifically to be reported, no evaluation of false positives was undertaken. In total, 23 false negatives pertaining to 18 different variants were observed over all three benchmarks and further investigated. One was caused by an operator manual transcription error (“EGFR p.(Glu746_Ala750delinsIlePro)” instead of “EGFR p.(Glu746-Ala750del)”), as the correct mutation could be observed in VCF files. Three were variants with expected frequencies below the LOD for those laboratories; therefore, these were not reported (LOD of 5% for all three laboratories), but they were found to be present in their sequencing data after manual inspection of their provided BAM files with IGV [28]. Thirteen variants were likely missed by the NGS workflows of participants, of which two had expected allelic frequencies close to the LOD of the concerned participants, one was classified as a variant of unknown significance (VUS) by a participant and was therefore not reported following their routine practices, and for the remaining ten, no discernable reason for omission could be determined. A more detailed overview of affected samples and variants is provided in Supplementary Table S7. Lastly, six variants were found to be missing because they had been artificially inserted in cell lines by the vendor by using a 2 kb genetic insertion cassette that resulted in an incompatibility between the benchmark material and gene panels used by five laboratories (three variants affecting two laboratories in benchmark 2017/1 and three variants affecting three laboratories in benchmark 2017/2). In those cases, primers employed in gene panels were positioned on either side of the genetic insertion cassette, resulting in amplicon lengths superior to those for which the panels were designed, thereby preventing amplification of the targeted regions. Therefore, these six missing variants were not taken into account for determining the global benchmark success rates and were omitted for the evaluation of affected laboratories. An overview of affected participants and variants is provided in Supplementary Table S8. An example illustrating this problematic is also provided in Supplementary Figure S1. To avoid further incompatibilities, samples for benchmark 2018/1 were specifically requested to the vendor to only include endogenous variants, limiting however the choice of variants and allelic frequencies available.

#### 2.2.4. Assessment of Allelic Frequencies for Evaluative Variants

For evaluative variants, the allelic frequencies of the reported variants were also considered for every participant and variant by means of calculating a Z-score that describes the deviation in a reported allelic frequency compared to the overall distribution of reported allelic frequencies by all participants for a particular variant (Table 3). In benchmark 2018/1, deviations in the reported allelic frequencies of participants were compared to the distribution of allelic frequencies of the peer group (consisting of participants using the same gene panel) and were only reported for participants belonging to peer groups of at least six participants. Using a maximum acceptance threshold of 3.0 for |Z|, 42 out of the 461 (9.11%) reported variants for which a Z-score was allocated, combined over all evaluative variants and participants for all benchmarks, received a Z-score citation. The proportion of Z-score citations with respect to the total number of observations for which a Z-score was allocated was in the same range over all three benchmarks with values of 7.69%, 10.53%, and 11.63% for benchmarks 2017/1, 2017/2, and 2018/1, respectively.

#### 2.2.5. Assessment of Repeatability for Evaluative Variants

The repeatability of reported variants across triplicates was evaluated for the evaluative variants for every benchmark and was generally high for all three benchmarks. For benchmark 2017/1, no repeatability issues were observed (i.e., all participants always reported an identified variant in all three replicates). For benchmark 2017/2, three participants exhibited repeatability issues. The first reported two variants in two out of three replicates, namely FLT3 p.(Asp835Tyr) in sample NGS-2017-005 and JAK2 p.(Val617Phe) in sample NGS-2017-006, and a third variant in one out of three replicates, namely KIT p.(Asp816Val) in sample NGS-2017-005. The second reported three variants in two out of three replicates, namely JAK2 p.(Val617Phe) and SF3B1 p.(Lys700Glu) in sample NGS-2017-006, and TP53 p.(Tyr220Cys) in sample NGS-2017-007. The third did not report any variants in one of the triplicates of sample 2017-007, as the sequencing did not meet the quality standards enforced in routine settings by the participant, and it was therefore omitted. For benchmark 2018/1, only one participant exhibited repeatability issues by reporting two variants in two out of three replicates, namely p.(Asn1784Thrfs*7) and p.(Lys1691Asnfs*15) in sample NGS-2018-001.

#### 2.2.6. Assessment of Standardization of Reporting Nomenclature Amongst Participants

Throughout all three benchmarks, a substantial variability in the nomenclature used for reporting variants by participants was observed. For instance, the BRAF p.(Val600Arg) variant was reported as p.(Val600Arg), p(Val600Arg), p.Val600Arg, p.V600R, p.Val600delinsArg, and p.V600delinsR in the 2017/1 benchmark (sample NGS-2017-002), despite explicit instructions to adhere to Human Genome Variation Society (HGVS) nomenclature (https://varnomen.hgvs.org/) [29]. Biological and clinical interpretations also varied widely between participants, with some reporting the pathogenicity level of the variant (with conflicting scales) or the evidence for a specific prognosis (or lack thereof) and others providing an in-depth review of the literature pertaining to the identified variants with both biological and clinical interpretations. Received data files also exhibited a lack of standardization. Sequencings were generally provided as one or two FASTQ files (depending on single- or paired-end reads), with one participant producing eight files per sequencing. Aligned sequencings were provided as one BAM and one BAI file, except for one participant who transferred one BAM file per targeted region resulting in over 400 files per sequencing. The presence of read groups and the ordering of reads in BAM files varied widely between participants. In one instance, the BAM file did not pass SAM validation by Picard 2.3.8 due to the “Proper pair” flag being set for unpaired reads. Another participant provided truncated BAM files. Lastly, VCF files similarly varied widely in employed data fields, as well as sizes, since some included a very limited set of filtered variants and others reported an exhaustive list of non-filtered variants.

## 3. Discussion

### 3.1. State-Of-The-Art and Performance of Clinical Tests Based on NGS for (Hemato-) Oncology within Belgium: An Overview

We present here the implementation of benchmarking trials (see Table 1) adopted with the ultimate aim of setting up an official EQA framework in Belgium for clinical tests based on NGS for (hemato-) oncology. DNA extracted from engineered cell lines mimicking cancer cells harboring sets of variants relevant to the targeted cancer types (see Table 2) was provided to participants with the explicit request to process samples according to their routine procedures for those cancer types and provide a list of detected variants of clinical relevance. Three benchmarks were performed during the period 2017–2018 pertaining to specific cancer types grouped in three activity domains: solid tumors (benchmark 2017/1), hematological malignancies (benchmark 2017/2), and *BRCA1/2* (benchmark 2018/1). Each benchmark was also coupled with a technical survey to obtain an overview of the state-of-the-art of employed methods for laboratories performing routine NGS for (hemato-) oncology in Belgium. The large majority of participants employed the Illumina technology, as was also reported recently in an international EQA [30], but this was in contrast to what has been reported in other international quality assessment trials, where the major platform used was Ion Torrent [31,32,33,34,35]. Employed sample types, genes panels, amplification approaches, bioinformatics software, and analytical thresholds varied substantially, as was also observed in other quality assessment trials [31,32,33,34,35]. Fewer participants reported employing in-house developed software or pipelines compared to what was reported in the USA [32]. Therefore, answers to the technical surveys underlined the variability of laboratory procedures currently existing within Belgium for clinical tests based on NGS for (hemato-) oncology, albeit dominated by the Illumina technology.

Despite the variability of laboratory procedures, an overall high performance was observed with success rates of 97.63%, 96.61%, and 93.06% for benchmarks 2017/1, 2017/2, and 2018/1, respectively (see Table 5). The vast majority of evaluative variants were reported by all participants for all three benchmarks, indicating high inter-laboratory repeatability (see Table 3). Moreover, the large majority of those were also reported for all three replicates per sample, indicating high intra-laboratory repeatability. These observations are in agreement with reports from other trials, and their comparisons suggest that NGS analyses in cancer-related genes in (hemato)-oncology carried out in Belgium have a satisfactory overall quality. Malapelle et al. showed 100% sensitivity at 5% and 10% allelic frequencies but lower at 1% [31], Merker et al. showed >98% sensitivity at 15% allelic frequency or higher [36], and Pisapia et al. reported 100% sensitivity in *KRAS* and *EGFR* genes at 5% and 10% allelic frequencies but lower at 1% allelic frequency or in other genes considered [33]. In a report published by Zhang et al., on 29 variants in cancer-related genes to be identified by 64 participants by NGS distributed over eight samples, 449 mistakes were recorded (201 false negatives, 222 false positives, and 26 slightly discordant results) [37]. The report from UK NEQAS for Molecular Genetics reference sample analysis, published by Richman et al., does not score directly their participants but describes results for variants identification in cancer-related genes from all methods combined, NGS or non-NGS, and advises maximizing opportunities to enroll in external quality assessment schemes [34]. The limited set of false negative observations in our data, corresponding to 23 cases over 18 different variants, could be reduced to the following causes: operator error (1), variants with allelic frequencies below (3) or close to (2), the LOD enforced by a participant for reporting, classification as a variant of unknown significance (1), undetectable due to an incompatibility between employed primers for gene panels and a 2 kb genetic insertion cassette in the benchmark material (6), or other undetermined reasons (10). False negatives due to incompatibility between employed gene panels and insertion cassette were omitted for an assessment of overall success rates and evaluation of individual participants, as these represent an artefact of the benchmark material rather than a real false negative observation. For all other types of false negatives, it is the responsibility of the individual laboratories to take actions as required in case of non-conformities and if necessary, undertake assay optimization procedures, which will be evaluated by BELAC during audits [19].

In accordance with published guidelines [38] and general practices in the field [20,32,34], reported allelic frequencies for different variants were not used to evaluate individual participants, but individual and global reports employed Z-scores and plots to inform laboratories on the distribution of allelic frequencies reported by other participants for every evaluative variant. Z-score citations were issued when the maximum acceptance value of |3| for the Z-score was surpassed, but they were provided for informative purposes only, and they are also listed in Table 3. Over all benchmarks, 9.11% of reported variants for which a Z-score could be calculated were cited for the Z-score.

The main issue identified in all three benchmarks consisted of discrepancies in the reporting of variants, either in the form of adherence to the correct nomenclature or biological/clinical interpretation. Cases of the former could easily be classified as ambiguous based on the formal HGVS nomenclature, and they were therefore accordingly addressed in the global and individual reports. However, both biological and clinical interpretations could not be evaluated in a straightforward fashion due to the lack of a clear standard of reporting to adhere to, but it was nevertheless shown to exhibit a large degree of variation upon manual inspection. Although interpretations were not considered for participant evaluation, they were included “as is” for informative purposes in the global reports for all benchmarks in light of the role of the benchmarks in establishing the state-of-the-art. Lastly, although data files were not considered for participant evaluation, a markedly large discrepancy was observed in data files despite the use of standardized formats such as BAM [39] and VCF [40], as was also reported by a recent international quality assessment effort [30].

### 3.2. Feasibility and Considerations for Implementing a Quality Assessment Framework for Clinical Tests Based on NGS for (Hemato-) Oncology

The set-up of the benchmarks proved to be effective to evaluate participants, whilst also providing both challenges to be addressed and opportunities for improvement. The choice of benchmark material provided to participants proved to be of major importance. We specifically employed DNA originating from cell lines, either as mixes of pure stocks or mixes of pure and mutated cell lines, obtained from a commercial vendor. This material was employed because it was readily available in sufficient quantity, homogenous, and easy to share across participants, and it allowed for the presence of multiple clinically relevant variants per sample reducing the overall costs [18], but it also presented some inherent limitations. Firstly, as was observed in benchmarks 2017/1 and 2017/2, artificially introduced variants can be incompatible with certain PCR-based amplification methods (Supplementary Figure S1). Therefore, for benchmark 2018/1, it was specifically requested to the vendor to only include endogenous variants to avoid any such incompatibilities; however, this reduced the choice of variants and allelic frequencies available. Secondly, the high number of extra variants that were present on top of the ordered variants in the cell lines that were used to create the DNA mixes rendered processing participant results, in particular biological and clinical interpretations, cumbersome and also unrealistic, because such highly mutated samples are not representative of tumors observed in routine clinical practice in the targeted cancer types. This effect was first observed in benchmark 2017/1, but it was still manageable due to the limited number of extra variants in the benchmark material that were present within regions of interest as defined by the convention of the Belgian National Institute for Health and Disability (INAMI/RIZIV) [17], resulting in four additional variants that had been validated by WES and consequently included as evaluative variants. However, this was much more pronounced for benchmark 2017/2 due to the particularly wide range of potential clinical targets resulting in 29 additional variants at clinically relevant positions. These variants were not validated by ddPCR and WES data were also not available; therefore, these variants were not used for evaluating participants but were provided in the global and individual reports for informative purposes only. For benchmark 2018/1, only two additional variants were reported by at least two-thirds of participants, which were also validated by prior WES and consequently included as evaluative variants. In other contexts such as hematological malignancies, this effect could even be exacerbated when using mixes of pure cell cultures carrying several variants. Thirdly, the benchmark material constituted an intermediate product compared to the routine workflows employed by most participants, which typically start from tissue samples such as biopsies and blood or FFPE samples, implying that the current set-up does not consider pre-sequencing steps (e.g., DNA extraction, purification…) and that the material does not exhibit some specificities observed in real samples, such as cancer cell density heterogeneity [41,42], intra-tumoral mutational landscape heterogeneity [43], and formalin-induced variants in FFPE samples [44]. However, real tumor samples, especially hematological ones, are difficult to impossible to source for quality assessment schemes given the health conditions of the patient(s) and the invasiveness of sampling substantial quantities of material to provide participants with a sufficient DNA yield. FFPE tumor samples are more readily available in tumor banks, but the intra-tumoral heterogeneity [36] and potentially low DNA yield due to small sample size(s) or degradation from intra-tumoral necrosis [45] also pose challenges for providing participants with material of sufficient quality. Moreover, both when using real tissue and FFPE samples, variants present in the sample would first need to be extensively validated to ensure their presence before sending out for any quality assessment scheme, whereas with the current solution, requested variants were certified by the vendor of the benchmark material. Additionally, few clinically relevant variants are expected for real tissue and FFPE samples, so that more samples would need to be sourced and validated to cover a wide set of clinically relevant variants. All these steps would increase overall costs considerably. Furthermore, since the start of the project, a greater choice of material is becoming available with more variants in a fully described genomic background. These reference quality standards can also be included in paraffin to mimic FFPE samples, allowing the incorporation of pre-analytical steps into the survey. Therefore, this approach serves as an attractive cost-effective solution that can be expanded upon as quality assessment schemes for NGS in clinical (hemato-) oncology continue to mature.

The central tenet of quality assessment is to evaluate participants based on an agreement between their results and a target value [18]. In the benchmarks presented here, the presence of ordered variants was validated using ddPCR and/or WES, and they were certified by the vendor of the benchmark material, thereby providing a well-characterized target value both qualitatively (i.e., the presence of a variant at a clinically relevant position) and quantitatively (i.e., its associated allelic frequency). Although quantitative information for the target values was also available, participant evaluation was based solely on the qualitative aspect, as also reported in other EQA schemes [46]. While the detection of a clinically relevant variant is important in routine clinical settings, its associated allelic frequency was not deemed necessary by consulted experts, and it is not required to be evaluated during quality assessment according to several published guidelines [38,47]. Therefore, the quantitative aspect was provided solely as additional information in the form of a Z-score that describes the deviation from the distribution of all reported allelic frequencies for each variant to allow self-evaluation by participants. However, recent guidelines published by the Food and Drug Administration now recommend reporting the variant allele frequency (VAF), because it can potentially help in delineating between germline and somatic variants and clonal diversity evaluation [48]. The VAF has also been shown to be promising for prognostic, diagnostic, and phenotypic prediction in hematologic malignancies [49,50]. The adaptation of future benchmarks to allow the evaluation of allelic frequency reporting will likely be required to match evolutions in clinical practice. In contrast to other quality assessment schemes such as the College of American Pathologists, participants were not provided a predefined list of positions for which they had to report the detected variant, but rather were explicitly requested to provide all variants of clinical relevance as defined in the Belgian convention [17] for the reimbursement of NGS tests. This approach was preferred, because it constitutes a more realistic scenario wherein participants were not given any a priori knowledge of positions of interest to investigate and focus on, but rather required them to report any variant of interest (as defined by the Belgian convention) present in an otherwise unknown sample representative for a certain cancer type, similarly to real-world conditions for samples they process routinely. Additionally, as the nomenclature to be used for variant reporting and other relevant information were clearly described, the evaluation of provided results was straightforward to implement for routine proficiency testing. We considered variants reported in at least one of three replicates by a participant as a positive hit, instead of requiring reporting in two or three replicates. This allowed an evaluation of intra-laboratory repeatability during the benchmarks, which was shown to be overall very good, but is not expected to be used in official proficiency testing, as is reported in other EQA schemes [20,36,37,46,51] in light of the additional cost for participants and the requirement for this type of analysis to be performed during method validation and IQC procedures [19]. An inherent limitation of our current set-up is that the target values consist solely of a positive target class, i.e., variants that need to be detected allowing discriminating between true positive and false negative observations. However, this does not consider a negative target class, i.e., wild-type positions allowing discriminating between true negative and false positive observations. Therefore, our set-up could potentially be expanded by also considering positions that contain wild-type nucleotides at clinically relevant positions that have been validated; however, this would constitute an additional layer of complexity on top of the current set-up. A simpler approach could consist of referring to the consensus of reported variants and consider those reported by a minority of participants as false positives.

As highlighted previously, discrepancies in the biological and/or clinical interpretation of variants was one of the main issues identified. Biological and/or clinical interpretation of variants, while being instrumental in translating the raw variant calling into patient treatment, proved difficult to implement as a criterion for participant evaluation. Firstly, a description of expected answers as well as definitions of biological versus clinical interpretation were limited and lacked clear guidelines, so that variant interpretation was left to the participant’s discretion, rendering it difficult to compare the different participant responses. Secondly, evaluation of biological and/or clinical interpretations would require a reference interpretation in order to compare participant’s answers, akin to a target value, for which a broad consensus is currently lacking; therefore, such an evaluation would require a substantial standardization effort including a broad panel of experts from different backgrounds. The variability observed in reported interpretations showcased the need for more in-depth and adapted studies in order to assess the level of standardization, for instance by way of interpretation-only quality assessments based on real test cases. To further standardize the biological classification and clinical interpretation of variants, a working group composed of members of the ComPerMed has been created with the task of taking up questions of variant interpretation standardization, resulting in the publication of guidelines to be used by laboratories performing NGS in routine cancer analysis [52]. These guidelines are also published on the BELAC website, reviewed regularly depending on the evolution of the field, and must be followed by all Belgian laboratories as legal obligation, thus guaranteeing a single interpretation for each variation in Belgium and forming a consensus statement in Belgium for variant interpretation (https://economie.fgov.be/sites/default/files/Files/Publications/files/Belac-FR/2-405NGS-FR.pdf). These new parameters will be incorporated into future external quality assessments, which will offer, in addition to a technical evaluation of the quality of NGS, an evaluation of biological and clinical interpretation of variants. Belgium has given itself the means to develop an external quality assessment program that is tailor-made and sustainable to control the overall quality of its laboratories, which can serve as an example for other countries wishing to develop a similar national approach in this field.

## 4. Materials and Methods

### 4.1. Benchmark Design

A general overview of benchmarks characteristics is provided in Table 1. For each benchmark, samples were specifically devised to harbor variants relevant to the cancer type(s) targeted by the benchmark. The first benchmark, referred to as ‘2017/1’, targeted colorectal (samples NGS-2017-001 and NGS-2017-002) and pulmonary (samples NGS-2017-003 and NGS-2017-004) carcinomas. The second benchmark, referred to as ‘2017/2’, targeted acute myeloblastic leukemia (sample NGS-2017-005), myeloproliferative neoplasia, and (pre-fibrotic) primary myelofibrosis (sample NGS-2017-006), and myelodysplastic syndromes (sample NGS-2017-007). The third benchmark, referred to as ‘2018/1’, targeted somatic variants in the *BRCA1* and *BRCA2* genes (samples NGS-2018-001, NGS-2018-002, and NGS-2018-003).

To mimic variants typically present in the targeted cancers, variant sets were selected to cover various genes and frequencies (5% to 50%), oncogenic and tumor suppressor genes (depending on cancer type), substitutions and indels, and these were based on the availability in the catalog of existing variants by the vendor of the material (Horizon Discovery, Cambridge, UK) and had to be located within regions of interest as defined in the convention of the INAMI/RIZIV for the reimbursement of NGS tests [17]. Samples were devised to carry multiple variants of interest to increase their informative value and also ensure that the cost of participation remained relatively limited [36]. A summarized overview of all samples and their targeted cancer types and genes for each benchmark is provided in Table 2, and an extensive overview of all ordered variants with their targeted transcript, protein, variant, and expected variant frequency is provided in Supplementary Table S6.

Sample material was ordered from Horizon Discovery (Cambridge, UK) as mixed genomic DNA from cell lines harboring the target variants at their specified frequencies. Samples for benchmarks 2017/1 and 2017/2 were produced from mixes of cell lines harboring endogenous variants of interest as well as cell lines with engineered variants, whereas samples for benchmark 2018/1 were produced from mixes of cell lines presenting endogenous variants only. Genomic DNA solution was selected as sample medium because it could be analyzed by all benchmark participants, it was guaranteed to be stable until 24 months after manufacturing, and it could be homogenized. The vendor guaranteed homogeneity of multiplexes and stability over time at 4 °C, and they also validated expected frequencies by digital-droplet PCR (ddPCR); these values are provided in Supplementary Table S6. Samples were sent to participants packaged as 17 µL or 20 µL of DNA solution at 50 ng/µL to allow for a minimal useable quantity of DNA of 250 ng per run and 100 ng of extra material. For benchmark 2017/1, the full 17 µL of sample received from the vendor was sent to each participant, whereas for benchmark 2017/2 and 2018/1, the material was received as samples of respectively 22 µL and 25 µL and was sent to participants as samples of respectively 17 µL and 20 µL, the remainder 5 µL being kept at the institute as a precautionary measure for confirmation analysis.

All Belgian laboratories performing genetic testing in the field of cancer (clinical biology, genetics, and anatomic pathology laboratories) accredited according to the ISO15189 standard [23] (or in the process of accreditation) were invited to participate in individual benchmarks corresponding to specific cancer types (solid tumors, hematological, and *BRCA1/2* genes). Participating laboratories were sent one DNA tube per multiplex packaged with cooling packs to maintain temperature between 2 and 8 °C during transport and an ESCORT iLOG Datalogger (LHM Instrumentation, Geel, Belgium) temperature logger, and they were requested to store samples between 2 and 8 °C. Samples were specifically demanded to be processed similarly to clinical samples of the simulated cancer types by integrating them in the participant’s routine analytical workflows. All samples for all three benchmarks were requested to be processed in triplicate to assess repeatability. Participants were asked to fill in a form for each sample to provide for each identified variant the following information: gene name, chromosome number and position, reference and observed nucleotides, description of the DNA-level variant following the Human Genome Variation Societies’ (HGVS) [53] nomenclature, RefSeq [54] mRNA-level reference number (NM), description of the protein-level variant following the HVGS nomenclature, RefSeq protein-level reference number (NP), variant type (missense, nonsense, frameshift), biological (related to tumorigenesis induction) and/or clinical (related to hindrance of treatment) interpretation, allelic frequency, and read coverage.

### 4.2. Technical Survey to Establish the State-Of-The-Art in Belgium

An accompanying survey was performed to collect the following information from participants regarding their employed workflows: types of variants normally reported by the laboratories (indels, SNPs, copy number variations (CNV), translocations), limit of detection (LOD), whether healthy tissue is also sequenced in conjunction with the tumor sample as a control and which particular tissue type, types of samples normally processed (FFPE, tumor tissue, frozen tissue), minimum required DNA quantity, NGS technology/vendor, NGS platform and flow-cell/chip reference, read lengths and whether reads are single- or paired-end, employed gene panel references, and employed bioinformatics workflows/tools (e.g., commercial solutions, in-house developed pipeline). Participating laboratories were also requested to provide bed/manifest files of the gene panels they evaluated, standard operation procedure (SOP) documentation for their entire sequencing workflow from sample to result, as well as BAM, FASTQ, and VCF files and a clinical report for each sample. Participants were given between five and six weeks to respond after sample shipment. For benchmarks 2017/1 and 2017/2, all requested data (variant calling results files, reports, raw data files, survey answers, and other quality documentation) were received via USB sticks provided to each participant. Data from one laboratory had to be fetched manually with a higher capacity hard disk due to the large size of generated data. For benchmark 2018/1, a more streamlined solution was implemented by developing a website where participants could encode their results and survey answers, while reports, data files, and other documents were returned via upload links generated by a FTP application. All FASTQ files produced by laboratories were uploaded to the Sequence Read Archive (SRA; https://www.ncbi.nlm.nih.gov/sra) under BioProject ID PRJNA659725.

### 4.3. Assessment of Benchmark Results

In accordance with standard procedures for proficiency testing [18,29,55,56], consensus from participant results was used to determine “evaluative variants”, i.e., variants that were used for the evaluation of participants and also assessment of global benchmark success rates. Therefore, evaluative variants were required to be reported by at least two-thirds of participants but also to be validated by either ddPCR or WES, and they had to be located within regions of interest as defined in the convention of the INAMI/RIZIV for the reimbursement of NGS tests [17]. Small variations in the benchmark design are discussed for each benchmark individually below.

For benchmark 2017/1, results were manually curated for operator mistakes such as obvious clerical errors in genomic positions, and protein-level variant names were standardized prior to analysis. Only variants present in regions covered by all participants, which was assessed with BEDTools 2.25.0 [57] and in-house developed scripts, were retained for further analysis. Evaluative variants consisted of 16 variants validated by ddPCR or WES by the vendor on the cell lines employed for production of samples (Table 3). BAM files were visually checked with IGV 2.4.10 [28] in case of unreported or misnamed variants. For each participant, and for each variant, the following statistics were considered: median allelic frequency over the three replicates (MRAF), median allelic frequency of all participants (MAF) calculated as the median of median values per triplicate, standard deviation (SD) of values for the allelic frequency reported by participants for a certain variant, and a Z-score calculated as Z = (MRAF − MAF)/SD. A maximum acceptance value for |Z| of 3.0 was enforced for evaluating the reported frequency of each variant per individual participant. For every variant, outliers, if present, were removed using Grubb’s tests, and normality over all participants was assessed both graphically through Q-Q plot and statistically by the Shapiro–Wilk test. ddPCR was performed on two samples (NGS-2017-003 and NGS-2017-004, retrieved back from the laboratory) to validate the presence of the variant EGFR p.(Gly719Ser) for one participant. To this end, the ddPCR assay EGFR p.(Gly719Ser) (dHsaMDV2010041, Bio-Rad Laboratories NV) was performed using a total of 10 ng of genomic DNA on the QX200TM Droplet Digital PCR System (Bio-Rad Laboratories NV, USA), as per the manufacturer’s protocol. Female human DNA (Promega) was used as negative control. Remaining material from the initially sent samples NGS-2017-003 and NGS-2017-004 was used as positive control. An individual report was provided to each participant in conjunction with an anonymized global report that was made available on the website of Sciensano [58].

For benchmark 2017/2, the same data curation as for benchmark 2017/1 was performed. Evaluative variants consisted of 16 variants validated by ddPCR by the vendor (Table 3). A total of 29 additional variants located within regions of interest as defined by the convention of the INAMI/RIZIV [17] but not validated by exome sequencing on the cell lines employed were reported by at least one participant, rendering it economically impractical to validate all of them by ddPCR. Therefore, these additional variants were considered as “informative variants”, i.e., they were included only as additional information in the individual and global reports but not used for evaluating participants. For each participant, and for each variant of interest, the same statistics and Z-score threshold were employed as for benchmark 2017/1. An individual report was provided to each participant, in conjunction with an anonymized global report that was made available on the website of Sciensano [59].

For benchmark 2018/1, the same data curation as for benchmarks 2017/1 and 2017/2 was performed. In total, 24 variants were confirmed based on prior exome sequencing of the employed cell lines or by ddPCR by the vendor (Table 3), of which six were reported by at least two-thirds of the participants and considered as evaluative. The 16 variants that were reported by no participant were not further analyzed, and the two variants that were reported by between one and two-thirds of participants were considered as informative variants. Evaluative variants unreported by certain participants were manually checked in their provided BAM files with IGV. For each participant, and for each variant of interest, the same statistics and Z-score threshold were employed as for benchmarks 2017/1 and 2017/2. Unlike in benchmarks 2017/1 and 2017/2, participants using the same gene panel were grouped into peer groups to allow comparison of the reported allelic frequency and the distribution of allelic frequencies. Z-scores were not allocated for participants belonging to small peer groups (N < 6). An individual report was provided to each participant, in conjunction with an anonymized global report that was made available on the website of Sciensano [60].

Overall success rates were defined for each benchmark as the number of correctly identified evaluative variants by a participant, without considering the reported allelic frequency, summed over all participants. Repeatability was not taken into account for the calculation of success rates, as any expected variant identified in at least one of the triplicates by a participant was considered correctly identified.

## 5. Conclusions

We presented three benchmarks of Belgian laboratories performing targeted NGS in routine cancer treatment on solid tumors, hematologic malignancies, and *BRCA1/2* genes, which took place between 2017 and 2018. These benchmarks aimed to establish the state-of-the-art of the field in Belgium and to develop the required expertise to implement proficiency testing at the Belgian level. The benchmarks showcased that despite most participants using the Illumina technology, a wide variety existed in routinely processed and employed sample types, genes panels, amplification approaches, bioinformatics software, and analytical thresholds. Nevertheless, laboratories performed generally well in all three benchmarks, demonstrating high levels of intra- and inter-laboratory repeatability with still some margin left for improvement. Our work highlights the numerous challenges faced when implementing EQA for NGS-centered oncology practice. Particularly, the employed material had a strong impact on the overall set-up and evaluation of quality assessment results. It should avoid incompatibilities with specific sequencing workflows and should be as close to “real-world” conditions as possible, whilst being informative at a cost that does not impede participation, and it should also be practical enough for sourcing sufficient material that can easily be distributed and for which the ground truth is defined. The biological and/or clinical interpretation of variants appears especially problematic and will benefit highly from standardization efforts and interpretation-only benchmarks. Combined, our work contributes towards the implementation of EQA schemes that will help improve quality of healthcare and ultimately benefit patient outcome.

## Figures and Tables

**Table 1:** General overview of benchmarks characteristics.

Benchmark	2017/1	2017/2	2018/1
**Targeted cancer types**	Colorectal carcinoma, pulmonary carcinoma	Acute myeloblastic leukemia, myeloproliferative neoplasia and (pre-fibrotic) primitive myelofibrosis, myelodysplastic syndromes	*BRCA1/BRCA2* genes
**Sample origin**	Mix of engineered and pure cell lines	Mix of engineered and pure cell lines	Mix of pure cell lines
**Participants**	16	15	12
**Variants considered for evaluation**	Variants reported by at least two-thirds of participants and validated by exome sequencing and/or ddPCR	Variants reported by at least two-thirds of participants and validated by ddPCR	Variants reported by at least two-thirds of participants and validated by exome sequencing and/or ddPCR
**Samples**	4	3	3
**Ordered variants**	12	16	6
**Total reported variants**	16	45	8
**Evaluative variants**	16 ^1^	16 ^2^	6 ^3^
**Validated variants (method)**	4 (WES), 12 (ddPCR)	16 (ddPCR)	8 (WES), 6 (ddPCR + WES)

^1^ The four additional reported variants on top of the 12 ordered variants, for a total of 16 reported variants, were validated by prior WES data, and were therefore also considered as evaluative. ^2^ The 29 additional variants on top of the 16 ordered variants for a total of 45 reported variants were not additionally validated by ddPCR and were therefore considered as informative only. ^3^ Of the eight reported variants, four had been ordered and were validated by ddPCR and WES, and four additional variants had been validated by WES, of which only two were reported by at least two-thirds of participants and therefore considered as evaluative. Abbreviations: ddPCR (digital-droplet polymerase chain reaction); WES (whole exome sequencing).

**Table 2:** Overview of samples and their targeted cancer types and genes to be evaluated by laboratories for each benchmark. All clinically relevant genes of the targeted cancer types are displayed, regardless of whether they were mutated or not in the sample. See also Supplementary Table S6 for a full overview of all samples with their targeted transcript and protein, as well as ordered variants and expected variant frequency.

Benchmark	Sample	Targeted Cancer Type(s)	Genes (Exons/Hotspots)
**2017/1**	NGS-2017-001/002	Colorectal carcinoma (advanced stage)	*BRAF* (exons 15 (codon 600))
			*KRAS* (exon 2 (codons 12,13), exon 3 (codons 59, 61), exon 4 (codons 117, 146))
			*NRAS* (exon 2 (codons 12,13), exon 3 (codons 59, 61), exon 4 (codons 117, 146))
	NGS-2017-003/004	Pulmonary carcinoma (advanced stage)	*BRAF* (exon 15 (codon 600))
			*EGFR* (exon 18 to 21)
			*ALK* (exon 22, exon 23, exon 25)
			*MET* (exon14 skipping)
**2017/2**	NGS-2017-005	Acute myeloblastic leukemia	*ASXL1* (exon 12)
			*CEBPA* (all exons)
			*DNMT3A* (exons 8 to 23)
			*FLT3* (exon 14, exon 15, exon 20 (codon 835))
			*IDH1* (exon 4 hotspot)
			*IDH2* (exon 4 hotspot)
			*KIT* (exon 8, exon 10, exon 17)
			*NPM1* (exon 11 (codon 288))
			*RUNX1* (all exons)
			*TET2* (exon 3, exons 9 to 11)
			*TP53* (exons 3 to 9)
			*WT1* (exon 7, exon 9)
	NGS-2017-006	Myeloproliferative neoplasia/(pre-fibrotic) primary myelofibrosis	*JAK2* (exon 12-F537_I546 ^1^, exon 14 (codon 617))
			*MPL* (exon 10 (codon 515))
			*CALR* (exon 9)
			*ASXL1* (exons 12)
			*EZH2* (all exons)
			*TET2* (exon 3, exons 9 to 11)
			*IDH1* (exon 4 hotspot)
			*IDH2* (exon 4 hotspot)
			*SRSF2* (exon 1 (codon 95))
			*SF3B1* (exon 14, exon 15)
	NGS-2017-007	Myelodysplastic syndromes	*SF3B1* (exon 14, exon 15)
			*TET2* (exon 3, exons 9 to 11)
			*SRSF2* (exon 1 (codon 95))
			*ASXL1* (exon 12)
			*DNMT3A* (exons 8 to 23)
			*RUNX1* (all exons)
			*U2AF1* (exon 2 (codon 34), exon 6 (codon 157))
			*TP53* (exons 3 to 9)
			*EZH2* (all exons)
**2018/1**	NGS-2018-001/002/003	/	*BRCA1* (all exons)
			*BRCA2* (all exons)

^1^ Hotspot region between F537 and I546 on exon 12 of JAK2.

**Table 3:** Participant results for evaluative variants per benchmark.

Benchmark	Sample	Gene	Variant (HGVS)	Median Allelic Frequency ^1^ (SD)	Z-Citations ^2^	Participant Success ^3^
**2017/1**	NGS-2017-001	*BRAF*	p.(Val600Glu)	13.21 (0.60)	1/16	16/16
		*KRAS*	p.(Gly13Asp)	32.94 (0.64)	1/16	16/16
		*NRAS*	p.(Gln61Lys)	21.55 (1.17)	1/16	16/16
	NGS-2017-002	*BRAF*	p.(Val600Arg)	11.26 (1.13)	1/16	16/16
		*KRAS*	p.(Ala146Thr) ^4^	20.07 (2.31)	3/15	15/16
		*NRAS*	p.(Gly12Asp)	19.42 (2.24)	1/16	16/16
	NGS-2017-003	*BRAF*	p.(Val600Lys)	48.50 (2.95)	0/16	16/16
		*EGFR*	p.(Glu746-Ala750del)	35.70 (2.89)	2/15	15/16
		*EGFR*	p.(Gly719Ser)	11.10 (1.38)	1/15	15/16
		*KRAS*	p.(Gly12Ala) ^4^	18.24 (1.36)	1/15	15/16
	NGS-2017-004	*BRAF*	p.(Val600Met)	19.73 (0.83)	2/16	16/16
		*EGFR*	p.(Gly719Ser)	3.73 (0.50)	0/12	12/16
		*EGFR*	p.(Leu858Arg)	38.13 (0.96)	1/16	16/16
		*EGFR*	p.(Thr790Met)	38.00 (1.10)	3/16	16/16
		*KRAS*	p.(Gly12Cys) ^4^	5.16 (0.42)	1/15	15/16
		*KRAS*	p.(Gly13Asp)	29.07 (0.95)	0/16	16/16
**2017/2**	NGS-2017-005	*TP53*	p.(Glu171*)	34.30 (1.76)	1/15	15/15
		*KIT*	p.(Asp816Val)	19.03 (1.67)	1/15	15/15
		*IDH2*	p.(Arg140Gln)	20.88 (1.11)	3/15	15/15
		*IDH1*	p.(Arg132Gly)	5.30 (0.60)	1/15	15/15
		*FLT3*	p.(Asp835Tyr) ^4^	11.46 (0.86)	1/14	14/15
	NGS-2017-006	*JAK2*	p.(Val617Phe)	21.00 (0.87)	2/15	15/15
		*IDH2*	p.(Arg172Ser)	30.95 (0.83)	1/14	14/15
		*IDH1*	p.(Arg132Ser)	11.05 (1.12)	1/14	14/15
		*SF3B1*	p.(Lys700Glu) ^4^	10.65 (1.24)	2/12	12/15
	NGS-2017-007	*SF3B1*	p.(Lys666Asn) ^4^	24.76 (2.63)	2/15	15/15
		*TP53*	p.(Ala161Asp)	47.45 (2.73)	2/14	14/15
		*TP53*	p.(Tyr220Cys)	5.12 (0.63)	1/13	13/15
**2018/1**	NGS-2018-001	*BRCA1*	p.(Arg1443*)	11 (0.22)	1/8	12/12
		*BRCA2*	p.(Asn1784Thrfs*7)	12 (0.13)	1/7	11/12
		*BRCA2*	p.(Lys1691Asnfs*15)	13 (0.23)	1/7	11/12
	NGS-2018-002	*BRCA2*	p.(Asn1784Thrfs*7)	20.7 (0.43)	1/7	11/12
	NGS-2018-003	*BRCA2*	p.(Asn1784Thrfs*7)	25.6 (0.61)	0/7	11/12
		*BRCA2*	p.(Ile2675Aspfs*6)	24 (0.68)	1/7	11/12

^1^ Median allelic frequency: median of allelic frequencies reported by all participants for a variant, with standard deviation. ^2^ Z-citations: number of participants that were cited for Z-score, i.e., surpassing the maximum acceptance value for |Z| of 3.0, of those for which a z-score could be calculated. ^3^ Participant success: number of participants that correctly reported the variant in at least one replicate. ^4^ Variants inserted by the vendor of the benchmark material, potentially causing incompatibility with primers used in the gene panels for gene amplification.

**Table 4:** Participant results for informative variants per benchmark.

Benchmark	Sample	Gene	Variant (HGVS)	Median Allelic Frequency ^1^ (SD)	Participant Identification ^2^
2017/2	NGS-2017-005	*TET2*	p.(Ser268*)	27.00 (1.21)	15/15
		*TP53*	p.(Ser215Gly)	46.34 (1.90)	14/15
		*ASXL1*	p.(Leu764Tyrfs*8)	40.20 (0.96)	13/15
		*RUNX1*	p.(Pro49Leu)	13.00 (1.04)	10/15
		*RUNX1*	p.(Met267Ile)	12.30 (2.20)	9/15
		*ASXL1*	p.(Gly646Trpfs*12)	8.00 (0.56)	6/15
		*TET2*	p.(Tyr867His)	51.10 (0.83)	5/15
		*TET2*	p.(Pro1723Ser)	48.81 (3.77)	4/15
		*ASXL1*	p.(Met1249Val)	7.79 (0.84)	4/15
		*TET2*	p.(Ile1762Val)	9.90 (0.59)	2/15
		*TET2*	p.(His1778Arg)	51.00 (0.15)	2/15
		*TP53*	p.(Pro72Arg)	98.50 (0.00)	1/15
		*DNMT3A*	p.(Arg729Trp)	2.80 (0.00)	1/15
		*CEBPA*	p.(His195_Pro196dup)	8.10 (0.00)	1/15
	NGS-2017-006	*ASXL1*	p.(Tyr591*)	10.10 (0.74)	13/15
		*ASXL1*	p.(Leu764Tyrfs*8)	68.00 (2.74)	13/15
		*TET2*	p.(Tyr867His)	69.82 (1.63)	5/15
		*TET2*	p.(Pro1723Ser)	67.74 (7.64)	5/15
		*TET2*	p.(Leu1721Trp)	20.30 (1.11)	2/15
		*TET2*	p.(Ile1762Val)	37.25 (22.28)	2/15
		*TET2*	p.(His1778Arg)	39.45 (21.83)	2/15
	NGS-2017-007	*EZH2*	p.(Cys539Arg)	21.19 (0.69)	11/15
		*TET2*	p.(Arg1261His)	26.70 (1.06)	8/15
		*TET2*	p.(Gln1084Pro)	41.10 (1.16)	5/15
		*TET2*	p.(Ile1762Val)	22.80 (2.15)	2/15
		*TP53*	p.(Pro72Arg)	53.70 (0.00)	1/15
		*TET2*	p.(Leu1721Trp)	3.15 (0.00)	1/15
		*EZH2*	p.(Asp146His)	7.80 (0.00)	1/15
		*DNMT3A*	p.(Arg729Trp)	2.80 (0.00)	1/15
2018/1	NGS-2018-002	*BRCA1*	p.(Asp435Tyr)	/ **^3^**	5/12
	NGS-2018-003	*BRCA1*	p.(Asp435Tyr)	/ **^3^**	5/12

^1^ Median allelic frequency: median of allelic frequencies reported by all participants for a variant, with standard deviation. ^2^ Participant identification: number of participants that correctly reported the variant in at least one replicate. ^3^ Refers to variants that were reported by too few participants of the same peer group to calculate a median allelic frequency.

**Table 5:** Summarized overview of participant success rates for all three benchmarks. Results are stratified by success rates. For each benchmark, the global success rate over all participants for all evaluative variants is indicated in bold.

Benchmark	Success Rate (Absolute Counts) ^1^	Participants ^2^
**2017/1**	100% (16/16 or 15/15 or 14/14) **^3^**	11 (68.75%)
	93.75% (15/16)	4 (25%)
	87.50% (14/16)	1 (6.25%)
	**97.63% (247/253)**	**16 (100%)**
**2017/2**	100% (12/12 or 11/11) **^3^**	11 (73.33%)
	91.67% (11/12)	3 (20%)
	75% (9/12)	1 (6.67%)
	**96.61% (171/177)**	**15 (100%)**
**2018/1**	100% (6/6)	11 (91.67%)
	17% (1/6)	1 (8.33%)
	**93.06% (67/72)**	**12 (100%)**

^1^ Success rate: number of variants correctly reported by a participant out of all evaluative variants. ^2^ Participants: number of participants having the specified success rate. ^3^ Some variants were omitted for determining success rates due to incompatibilities between the used gene panels due to an insertion sequence being used to generate the variant in the benchmark material (see Supplementary Figure S1).

## Data Availability

All raw fastq files have been submitted to the NCBI Sequence Read Archive (SRA) under BioProject accession number PRJNA659725.

## Supplementary Materials

The following are available online at https://www.mdpi.com/2072-6694/12/11/3180/s1, Figure S1: Illustration of a variant not being detected due to a genetic insertion cassette resulting in an incompatibility between the benchmark material and certain gene panels. Table S1: Overview of employed sample types reported in the different benchmarks, Table S2: Overview of bioinformatics software reported being used in routine, Table S3: Overview of minimum reads depth and allelic frequencies for a variant to be reported by participants, Table S4: Overview of employed genes panels reported in the different benchmarks, Table S5: Overview of minimal DNA quantity required for analysis reported in the different benchmarks, Table S6: Overview of all ordered variants and corresponding relevant sequence information, Table S7: Overview of missed variants due to reasons other than incompatibilities between the variant inserted by an endogenous insertion cassette and gene panels employed by some participants, Table S8: Overview of missed variants due to incompatibilities between the variant inserted by an endogenous insertion cassette and gene panels employed by some participants.
