# Supplementary material for: NGS for (Hemato-) Oncology in Belgium: Evaluation of Laboratory Performance and Feasibility of a National External Quality Assessment Program
Source: Cancers (Basel). 2020 Oct 29;12(11):3180. doi: 10.3390/cancers12113180 (PMC7692129; doi:10.3390/cancers12113180)
Supplement: Supplementary file 1 [file cancers-12-03180-s001.pdf]

cancers

MDPI

Article

NGS for (hemato-)oncology in Belgium: evaluation of laboratory performance and feasibility of a national external quality assessment program

Thomas Delcourt [1], Kevin Vanneste [1], Mohamed Rida Soumali [2], Wim Coucke [2], Vanessa Ghislain [2], Aline Hebrant [3], Els Van Valckenborgh [3], Sigrid C. J. De Keersmaecker [1], Nancy H. Roosens [1], Philippe Van De Walle [2], Marc Van Den Bulcke [3] and Aline Antoniou [2,*]

[1] Transversal activities in Applied Genomics, Sciensano, 1050 Brussels, Belgium; Thomas.Delcourt@sciensano.be (T.D.); Kevin.Vanneste@sciensano.be (K.V.); Sigrid.DeKeersmaecker@sciensano.be (S.C.J.D.K.); Nancy.Roosens@sciensano.be (N.H.R.)

[2] Quality of Laboratories, Sciensano, 1050 Brussels, Belgium; MohamedRida.Soumali@sciensano.be (M.R.S.); Wim.Coucke@sciensano.be (W.C.); Vanessa.Ghislain@sciensano.be (V.G.); Philippe.VanDeWalle@sciensano.be (P.V.D.W.)

[3] Cancer Centre, Sciensano, 1050 Brussels, Belgium; Els.VanValckenborgh@sciensano.be (E.V.V.); Aline.Hebrant@sciensano.be (A.H.); Marc.VanDenBulcke@sciensano.be (M.V.D.B.)

* Correspondence: aline.antoniou@sciensano.be

Received: date; Accepted: date; Published: date

Simple Summary: In recent years, high-throughput sequencing has been routinely used by medical laboratories to search for somatic mutations in (hemato-)oncology as diagnostic, prognostic or therapeutic markers in various cancers. Since 2016, Belgium has developed a comprehensive program to facilitate the implementation of this technology in the national healthcare system, requiring, among others, an external quality assessment (EQA) of laboratories using this technology. Three benchmarking trials were organized between 2017 and 2018, covering different pathologies to establish the state of the art of the current practices of the Belgian laboratories and prepare future EQA. This study has highlighted areas of improvement for laboratories and will serve as a baseline for the establishment of a sustainable national EQA.

Abstract: Next-generation sequencing (NGS) is being integrated into routine clinical practice in the field of (hemato-) oncology to search for variants with diagnostic, prognostic, or therapeutic value at potentially low allelic frequencies. The complex sequencing workflows used require careful validation and continuous quality control. Participation in external quality assessments (EQA) helps laboratories evaluate their performance and guarantee the validity of tests results with the ultimate goal of ensuring high-quality patient care. Here, we describe three benchmarking trials performed during the period 2017–2018 aiming firstly at establishing the state-of-the-art and secondly setting up a NGS-specific EQA program at the national level in the field of clinical (hemato-) oncology in Belgium. DNA samples derived from cell line mixes and artificially mutated cell lines, designed to carry variants of clinical relevance occurring in solid tumors, hematological malignancies, and *BRCA1*/*BRCA2* genes, were sent to Belgian human genetics, anatomic pathology, and clinical biology laboratories, to be processed following routine practices, together with surveys covering technical aspects of the NGS workflows. Despite the wide variety of platforms and workflows currently applied in routine clinical practice, performance was satisfactory, since participating laboratories identified the targeted variants with success rates ranging between 93.06% and 97.63% depending on the benchmark, and few false negative or repeatability issues were identified. However, variant reporting and interpretation varied, underlining the need for further standardization. Our approach showcases the feasibility of developing and implementing EQA for routine clinical practice in the field of (hemato-) oncology, while highlighting the challenges faced.

Keywords: next-generation sequencing; hemato-oncology; oncology; external quality assessment; cancer

Supplementary

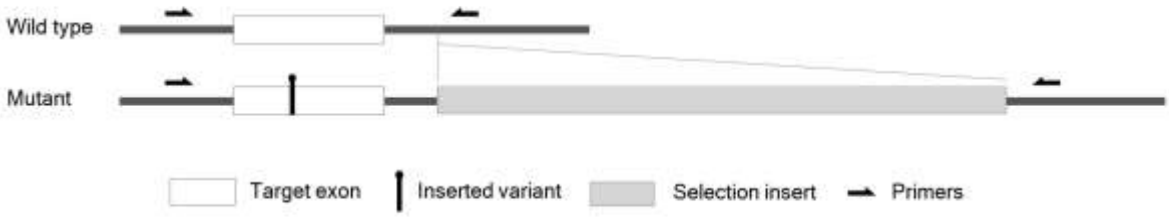

Figure S1. Illustration of a variant not being detected due to a genetic insertion cassette resulting in an incompatibility between the benchmark material and certain gene panels. The wild type sequence indicates the expected position of the primers with respect to the target exon. In this configuration, the distance between primers allows for a correct PCR amplification. The mutant sequence, however, contains the inserted variant as well as a selection sequence of about 2 kb between the target exon and the right-most primer. In this configuration, primers are too far apart to allow for a correct PCR amplification so that the inserted variant cannot be detected. An overview of all six variants missed due to such incompatibilities is listed in Supplementary Table 7.

Table S1. Overview of employed sample types reported being analyzed in routine in the different benchmarks.

Benchmark	Sample type	# participants [1]
2017/1	FFPE tissue	16
	Cytological liquids	3
	Fresh tissue	2
	Frozen tissue	1
	Blood	1
	Swabs	1
	Circulating tumor DNA	1
2017/2	Bone marrow	15
	Blood	14
	Frozen tissue	4
	FFPE tissue	1
	Biopsies and biological fluids	1
2018/1	FFPE tissue	11
	Blood	2
	Frozen tissue	1
	Cytology	1

[1] Number of times each sample matrix was reported to be used by a participant for each benchmark.

Table S2. Overview of bioinformatics software reported being used in routine.

Benchmark	Bioinformatics software (vendor)	# participants [1]
2017/1	VariantStudio (Illumina)	6
	Miseq Reporter (Illumina)	6
	SeqNext (JSI)	5
	NextGene (Softgenetics))	2
	Sophia DDM (Sophia Genetics)	2
	Torrent suite + TVC (Ion Torrent)	1
	Genome Browser (Golden Helix)	1
	BWA + GATK + Annovar (open-source)	1
2017/2	Sophia DDM (Sophia Genetics)	5
	VariantStudio (Illumina)	4
	SeqNext (JSI)	4
	NextGene (Softgenetics)	1
	Biomedical Genomics Workbench (Qiagen)	1
	CLC Genomics Workbench (Qiagen)	1
	QCI interpret (Qiagen)	1
	Agilent SureCall (Agilent)	1
	BWA + GATK + Annovar (open-source)	1
	FastQC + BWA + Samtools + Picardtools + Genome Analysis Toolkit (open-source)	1
2018/1	SeqNext (JSI)	7
	Sophia DDM (Sophia Genetics)	2
	Software Qiagen [2]	1
	DNA amplicon plugin (Illumina Basespace)	1
	CLC Bio + in-house scripts	1

[1] Number of times each bioinformatics software was reported to be used by a participant for each benchmark.

[2] Exact software was not specified.

Table S3. Overview of minimum reads depth and allelic frequencies for a variant to be reported by participants.

Benchmark	Variant type	Allelic frequency LOD	Read depth LOD	# participants [1]
2017/1	NA	NA	300	2
		NA	500	7
		NA	1000	7
	SNV	1	NA	3
		2.5	NA	1
		3	NA	1
		4	NA	1
		5	NA	10
	Indel <50bp	1	NA	2
		2.5	NA	2
		3	NA	1
		4	NA	1
		5	NA	10
2017/2	SNV	2	40	1
		2.5	/	1
		5	300	3
		5	500	6
		5	/	3
		1-5	/	1
	Indel <50bp	2	40	1
		5	300	3

		5	500	5
		5	/	4
		1-5	/	1
		/	/	1
	Indel 50 - 150 bp	2	40	1
		5	500	1
	Indel 150-1kb	5	300	1
		5	/	1
2018/1	SNV	4	500	1
		4	1000	1
		5	200-500	1
		5	300	4
		5	1000	2
		10	100	1
		10	300	1
		10	500	1
	Indel	4	500	1
		4	1000	1
		5	200-500	1
		5	300	4
		5	1000	2
		10	100	2
		10	500	1
	CNV	5	300	1
	Translocation	5	300	1

[1] Number of participants reporting this threshold.

/: No minimum threshold reported by participant.

Abbreviations: NA (Not applicable - for benchmark 2017/1, per-variant type thresholds were not evaluated for allelic frequency); SNV (Single Nucleotide Variant); Indel (Insertion/deletion); CNV (Copy Number Variation)

Table S4. Overview of employed genes panels reported in the different benchmarks

**Benchmark**	**Targets panel (vendor)**	**# participants** [1]
2017/1	Trusight Tumor 26 (Illumina)	3
	Trusight tumor 15 (Illumina)	2
	BRCA Tumor (MASTR Plus)	1
	Ion AmpliSeq Cancer Hotspot panel v2 (ThermoFisher)	1
	Ion AmpliSeq Colon and Lung Research Panel v2 (ThermoFisher)	1
	TruSeq Amplicon Cancer Panel 48 (Illumina)	1
	QIAact Actionable Insights Tumor Panel (Qiagen)	1
	Tumor Hotspot (MASTR Plus)	1
	Custom panel designed via unreporter software/provider	8
	TruSeq Custom Amplicon INCa panel (Illumina)	1
	TruSeq Custom Amplicon BRCA panel (Illumina)	1
	TruSeq Custom Amplicon panel (Illumina)	1
	Custom Ion AmpliSeq Panel "Gyneco" (ThermoFisher)	1
	Custom Ion AmpliSeq Panel "Colon-lung" (ThermoFisher)	1
2017/2	TruSight Myeloid Sequencing panel (Illumina)	7
	Human Myeloid Neoplasms panel (Qiagen)	1
	AmpliSeq Oncomine Myeloid (Thermo Fisher)	1
	xGen Acute Myeloid Leukemia Cancer Panel (IDT)	1
	Haloplex Custom panel (Agilent)	2
	TruSeq Custom Amplicon Low input (Illumina)	1
	Nextera XT Custom panel (Illumina)	1
	GeneRead Custom panel v2 (Qiagen)	1
2018/1	BRCA MASTR Plus Dx (Multiplicom-Agilent)	8
	AmpliSeq for Illumina BRCA panel (Illumina)	2
	GeneRead QIAact BRCA1 /2 panel (Qiagen)	1
	NimbleGen SeqCap EZ Choice custom panel (Roche)	1

[1] Number of times each genes panel was reported to be used by a participant for each benchmark.

Table S5. Overview of minimal DNA quantity required for analysis reported in the different benchmarks

Benchmark	DNA quantity (ng)	# participants [1]
2017/1	≤50	10
	51-100	2
	101-200	2
	201-300	1
2017/2	20	2
	30	1
	40	2
	50	7
	185	1
	200	1
	250	1
	1000	1
2018/1	≤10	2
	11 - 50	2
	51-100	3
	550	1
	NA	4

1 Number of times each minimum DNA quantity was reported to be used by a participant for each benchmark.

cancers

Table S6. Overview of all ordered variants and corresponding relevant sequence information.

Sample[1]	Gene	NM[2]	NP[3]	Variant (protein)[4]	Variant (DNA)[5]	Expected frequency[6]	ddPCR frequency[7]
NGS-2017-001	*BRAF*	NM_004333.5	NP_004324.2	p.(Val600Glu)	c.1799T>A	12.6-15.4%	12.59
NGS-2017-001	*KRAS*	NM_033360.3	NP_203524.1	p.(Gly13Asp)	c.38G>A	31.5-38.5%	32.00
NGS-2017-001	*NRAS*	NM_002524.5	NP_002515.1	p.(Gln61Lys)	c.181C>A	20.25-24.75%	21.53
NGS-2017-002	*BRAF*	NM_004333.5	NP_004324.2	p.(Val600Arg)	c.1798_1799GT>AG	11.25-13.75%	11.58
NGS-2017-002	*KRAS*	NM_033360.3	NP_203524.1	p.(Ala146Thr)	c.436G>A	18.45-22.55%	20.72
NGS-2017-002	*NRAS*	NM_002524.5	NP_002515.1	p.(Gly12Asp)	c.35G>A	18.45-22.55%	20.70
NGS-2017-003	*BRAF*	NM_004333.5	NP_004324.2	p.(Val600Lys)	c.1798_1799GT>AA	47.7-58.3%	50.70
NGS-2017-003	*EGFR*	NM_005228.4	NP_005219.2	p.(Glu746-Ala750del)	c.2235_2249del15	32.76-40.04%	33.60
NGS-2017-003	*KRAS*	NM_033360.3	NP_203524.1	p.(Gly12Ala)	c.35G>C	19.8-24.2%	18.27
NGS-2017-004	*BRAF*	NM_004333.5	NP_004324.2	p.(Val600Met)	c.1798G>A	18.9-23.1%	19.40
NGS-2017-004	*EGFR*	NM_005228.4	NP_005219.2	p.(Thr790Met)	c.2369C>T	36.9-45.1%	38.60
NGS-2017-004	*KRAS*	NM_033360.3	NP_203524.1	p.(Gly12Cys)	c.34G>T	4-6%	5.07
NGS-2017-005	*TP53*	NM_001276761.1	NP_001263690.1	p.(Glu171*)	c.511G>T	27.0-37.5%	34.00%
NGS-2017-005	*KIT*	NM_001093772.1	NP_001087241.1	p.(Asp816Val)	c.2447A>T	12.5-27.5%	19.90%
NGS-2017-005	*IDH2*	NM_002168.3	NP_002159.2	p.(Arg140Gln)	c.419G>A	18.0-25.3%	19.00%
NGS-2017-005	*IDH1*	NM_005896.3	NP_005887.2	p.(Arg132Gly)	c.394C>G	4.0-6.3%	5.30%
NGS-2017-005	*FLT3*	NM_004119.2	NP_004110.2	p.(Asp835Tyr)	c.2503G>T	9.0-12.7%	11.50%
NGS-2017-006	*JAK2*	NM_004972.3	NP_004963.1	p.(Val617Phe)	c.1849G>T	19.0-38.5%	20.40%
NGS-2017-006	*IDH2*	NM_002168.3	NP_002159.2	p.(Arg172Ser)	c.516G>T	27.0-40.0%	30.20%
NGS-2017-006	*IDH1*	NM_005896.3	NP_005887.2	p.(Arg132Ser)	c.394C>A	9.0-13.3%	11.00%
NGS-2017-006	*SF3B1*	NM_012433.3	NP_036565.2	p.(Lys700Glu)	c.2098A>G	11.3-16.7%	12.00%
NGS-2017-007	*SF3B1*	NM_012433.3	NP_036565.2	p.(Lys666Asn)	c.1998G>T	22.5-27.5%	27.20%
NGS-2017-007	*TP53*	NM_001276761.1	NP_001263690.1	p.(Ala161Asp)	c.482C>A	40.5-49.5%	48.90%
NGS-2017-007	*TP53*	NM_001276761.1	NP_001263690.1	p.(Tyr220Cys)	c.659A>G	4.0-6.0%	4.40%
NGS-2018-001	*BRCA1*	NM_007294.3	NP_009225.1	p.(Arg1443Ter) (R1443*)	c.4327C>T	9.0-11.0%	11.10%
NGS-2018-001	*BRCA2*	NM_000059.3	NP_000050.2	p.(Lys1691Asnfs*15) (K1691fs)	c.5073del	9.0-11.0%	10.20%
NGS-2018-002	*BRCA1*	NM_007294.3	NP_009225.1	p.(Lys820Glu)	c.2458A>G	18.0-22.0%	20.80%
NGS-2018-002	*BRCA2*	NM_000059.3	NP_000050.2	p.(Asn1784Thrfs*7) (N1784fs)	c.5351del	18.0-22.0%	19.40%
NGS-2018-003	*BRCA1*	NM_007294.3	NP_009225.1	p.(Pro871Leu)	c.2612C>T	45.0-55.0%	49.20%

NGS-2018-003	*BRCA2*	NM_000059.3	NP_000050.2	p.I2675fs*6 (I2675fs)	c.8021_8022insA	22.5-27.5%	25.20%

[1] Sample names. Samples NGS-2017-001, NGS-2017-002, NGS-2017-003 and NGS-2017-004 were used for benchmark 2017/1; samples NGS-2017-005, NGS-2017-006, NGS-2017-007 for benchmark 2017/2; and samples NGS-2018-001, NGS-2018-002 and NGS-2018-003 for benchmark 2018/1.

[2] NCBI RefSeq transcript reference number.

[3] NCBI RefSeq protein reference number.

[4] Protein-level variant name following the HGVS nomenclature.

[5] DNA-level variant name following the HGVS nomenclature.

[6] Expected frequency based on the provider’s indications.

[7] Variant frequency obtained by ddPCR validation.

cancers

Table S7. Overview of missed variants due to reasons other than incompatibilities between the variant inserted by an endogenous insertion cassette and gene panels employed by some participants

Sample	Gene	Variant (HGVS)[1]	Expected AF[2]	# participants[3]	Reason
NGS-2017-003	*EGFR*	p.(Glu746-Ala750del)	35.7	1	Clerical error
NGS-2017-003	*EGFR*	p.(Gly719Ser)	11.1	1	Workflow
NGS-2017-004	*EGFR*	p.(Gly719Ser)	3.7	4	LOD (3), workflow (1)
NGS-2017-006	*IDH2*	p.(Arg172Ser)	31.0	1	Workflow
NGS-2017-006	*IDH1*	p.(Arg132Ser)	11.1	1	Workflow
NGS-2017-006	*SF3B1*	p.(Lys700Glu)	10.7	1	Workflow
NGS-2017-007	*TP53*	p.(Ala161Asp)	47.5	1	Workflow (VUS)
NGS-2017-007	*TP53*	p.(Tyr220Cys)	5.1	2	Workflow (LOD)
NGS-2018-001	*BRCA2*	p.(Asn1784Thrfs*7)	12.0	1	Workflow
NGS-2018-001	*BRCA2*	p.(Lys1691Asnfs*15)	13.0	1	Workflow
NGS-2018-002	*BRCA2*	p.(Asn1784Thrfs*7)	20.7	1	Workflow
NGS-2018-003	*BRCA2*	p.(Asn1784Thrfs*7)	25.6	1	Workflow
NGS-2018-003	*BRCA2*	p.(Ile2675Aspfs*6)	24.0	1	Workflow

[1] Protein-level variant name following the HGVS nomenclature.

[2] Expected allelic frequency of the variant.

[3] Number of laboratories that have not reported the variant.

Abbreviations: LOD (limit of detection); VUS (variant of unknown significance).

Table S8. Overview of missed variants due to incompatibilities between the variant inserted by an endogenous insertion cassette and gene panels employed by some participants.

Benchmark	Participant	Incompatible variant	Sample
	Participant 1	KRAS p.(Ala146Thr)	NGS-2017-002
2017/1	Participant 2	KRAS p.(Gly12Ala)	NGS-2017-003
	Participant 2	KRAS p.(Gly12Cys)	NGS-2017-004
	Participant 3	FLT3 p.(Asp835Tyr)	NGS-2017-005
2017/2	Participant 4	SF3B1 p.(Lys700Glu)	NGS-2017-006
	Participant 5	SF3B1 p.(Lys700Glu)	NGS-2017-006

© 2020 by the authors. Submitted for possible open access publication under the terms and conditions of the Creative Commons Attribution (CC BY) license (http://creativecommons.org/licenses/by/4.0/).
